# Mucoadhesive Electrospun Nanofiber-Based Hybrid System with Controlled and Unidirectional Release of Desmopressin

**DOI:** 10.3390/ijms23031458

**Published:** 2022-01-27

**Authors:** Mai Bay Stie, Johan Ring Gätke, Ioannis S. Chronakis, Jette Jacobsen, Hanne Mørck Nielsen

**Affiliations:** 1 Department of Pharmacy, University of Copenhagen, Universitetsparken 2, 2100 Copenhagen, Denmark; mai.bay.stie@sund.ku.dk (M.B.S.); kjn376@alumni.ku.dk (J.R.G.); jette.jacobsen@sund.ku.dk (J.J.); 2 Center for Biopharmaceuticals and Biobarriers in Drug Delivery, Department of Pharmacy, University of Copenhagen, Universitetsparken 2, 2100 Copenhagen, Denmark; 3 DTU-Food, Technical University of Denmark, Kemitorvet, B202, 2800 Kongens Lyngby, Denmark; ioach@food.dtu.dk

**Keywords:** sublingual delivery, biopharmaceuticals, peptide drug delivery, electrospinning, mucoadhesion, ex vivo flow retention model

## Abstract

The sublingual mucosa is an attractive route for drug delivery, although challenged by a continuous flow of saliva that leads to a loss of drug by swallowing. It is of great benefit that drugs absorbed across the sublingual mucosa avoid exposure to the harsh environment of the gastro-intestinal lumen; this is especially beneficial for drugs of low physicochemical stability such as therapeutic peptides. In this study, a two-layered hybrid drug delivery system was developed for the sublingual delivery of the therapeutic peptide desmopressin. It consisted of peptide-loaded mucoadhesive electrospun chitosan/polyethylene oxide-based nanofibers (mean diameter of 183 ± 20 nm) and a saliva-repelling backing film to promote unidirectional release towards the mucosa. Desmopressin was released from the nanofiber-based hybrid system (approximately 80% of the loaded peptide was released within 45 min) in a unidirectional manner in vitro. Importantly, the nanofiber–film hybrid system protected the peptide from wash-out, as demonstrated in an ex vivo flow retention model with porcine sublingual mucosal tissue. Approximately 90% of the loaded desmopressin was retained at the surface of the ex vivo porcine sublingual mucosa after 15 min of exposure to flow rates representing salivary flow.

## 1. Introduction

Biopharmaceuticals—e.g., peptides and proteins—are the fastest growing group of drugs [1]. Peptides and proteins are highly potent and specific, but suffer from a low physicochemical stability; poor absorption across biological membranes; and, as a consequence, low bioavailability [2]. The primary route of administration of peptides and proteins is therefore via injections, which is inconvenient and often associated with poor patient compliance; new approaches for non-injectable formulations of peptides and proteins are therefore needed. The sublingual route is an attractive, non-invasive alternative site of administration as the mucosa is non-keratinized, highly vascularized, and consists of only 8–12 cell layers and thus has a reasonably low thickness (100–200 µm) compared to the much thicker buccal mucosa [3]. Furthermore, drugs delivered via the oral cavity avoid the harsh environment of the gastro-intestinal tract and bypass the hepatic first-pass metabolism. These factors are beneficial for drugs, which are prone to enzymatic degradation and sensitive to the low pH of the stomach [4]. Formulations for sublingual administration can easily be handled by the patient as the ventral side of the tongue is readily accessible and the quick removal of the formulation is possible if needed. Further, such formulations can be administered without water, which is beneficial for patient groups with swallowing difficulties—e.g., the elderly or small children [5].

Sublingual administration of peptides and proteins is challenged by a continuous flow of saliva (total volume of saliva of 0.5–2 L/day) that may lead to the swallowing of the drug or drug delivery system and the subsequent degradation of the drug in the gastro-intestinal tract [6]. Even if not swallowed, the dilution of drug in the saliva impairs absorption by decreasing the amount of drug available at the site of absorption and thus also the drug concentration gradient across the mucosal epithelium, the most significant biological barrier to the absorption of the drug. Through a rational design, mucoadhesive drug delivery systems can protect the drug by encapsulation, retain the drug at the site of application for a prolonged period by controlled drug release, and thus not only increase, but also maintain the drug concentration gradient across the tissue, leading to the improved absorption of drug and therefore higher systemic bioavailability.

Electrospun nanofibers are especially suitable for topical administration on, e.g., the sublingual mucosa because of their high surface area to volume ratio and tunable mechanical properties, which can make them flexible enough to bend to the curved mucosal surfaces in the mouth. We have previously demonstrated a robust protocol for the electrospinning of biocompatible chitosan/polyethylene oxide (PEO) nanofibers [7] and demonstrated their suitability for sublingual administration because of their mucoadhesive properties [8]. The use of electrospun nanofibers has been explored for the oromucosal delivery of peptides and proteins, including insulin [9,10,11], FITC-labelled albumin [12], and lysozyme [13]. Reports aiming to achieve peptide delivery by the sublingual route are rare [9].

The therapeutic peptide desmopressin is used for the treatment of, amongst others, nocturnal enuresis, diabetes insipidus and hemophilia A. This peptide is currently administered either as a solution by intravenous or subcutaneous injection, as a nasal spray, or as a freeze-dried sublingual tablet. The bioavailability of desmopressin after sublingual administration of a freeze-dried fast-dissolving tablet is, however, reported to be only ~0.25% [14], leaving room for improvement. Desmopressin is a relatively small peptide; it is modified from the structure of vasopressin (an endogenous hormone with a half-life in plasma of 10–35 min) to display a higher stability (half-life in plasma ~160 min) and 10 times higher antidiuretic potency and 1500 times lower vasoconstricting potency compared to its natural analogue [15].

In this work, a combination of mucoadhesive electrospun chitosan/PEO nanofibers that facilitate the controlled release of desmopressin and a local high concentration at the site of application, and a water-repelling backing film that ensures unidirectional release and prevents the wash-out of peptide by saliva, were assessed for the sublingual delivery of the therapeutic peptide desmopressin. The protection of desmopressin from wash-out by saliva subsequent to the sublingual administration of the nanofiber–film hybrid system was also evaluated ex vivo.

## 2. Results and Discussion

### 2.1. Clinically Relevant Doses of Desmopressin Loaded in Nanofiber–Film Hybrid System

**Figure 1:**
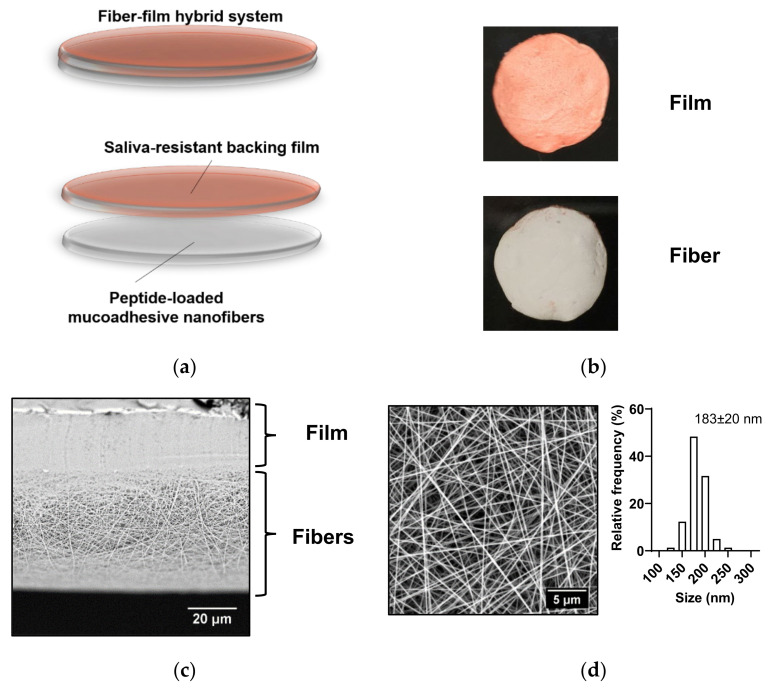
Properties of the desmopressin-loaded nanofiber–film hybrid system. (**a**) Schematic representation of the nanofiber–film hybrid system. (**b**) Image of a 10 mm disc of the two-sided nanofiber–film hybrid system. (**c**) Cross-section of peptide-loaded nanofiber–film hybrid system visualized by SEM. (**d**) Desmopressin-loaded chitosan/PEO nanofibers visualized by SEM. The size distribution of the nanofibers is given. The diameter is presented as mean ± SD. *N* = 3, *n* = 100, where *N* is the number of individual batches of nanofibers produced, and *n* is the number of nanofibers measured per batch.

A two-layered hybrid drug delivery system, which consisted of a mucoadhesive electrospun chitosan-based nanofiber mat and a saliva-repelling backing film (Figure 1a), was developed. It is important to note that the peptide delivery system is considered biocompatible, as all selected excipients are biocompatible, and the electrospun nanofibers were prepared using a minimum amount of acetic acid (0.7% (*w*/*w*)). The therapeutic peptide desmopressin was encapsulated within the nanofibers by co-electrospinning. Then, a saliva-repelling backing layer was sprayed onto the fiber mat as a thin film based on ethyl-cellulose. The food coloring iron oxide, a red pigment insoluble in water, was also included in the film to provide a visual discrepancy in the orientation of the nanofiber–film hybrid system (Figure 1b). As visualized by scanning electron microscopy (SEM), close connection between the fibers and the thin coherent film was achieved (Figure 1c). The peptide-loaded nanofibers were smooth, uniform, and without artifacts such as beading (Figure 1d), with an average diameter of 183 ± 20 nm (mean ± standard deviation (SD)) and with a narrow size distribution. The average diameter of the nanofibers is similar (168 ± 38 nm) to that of electrospun chitosan/PEO nanofibers prepared under the same conditions without the peptide [8].

The loading of desmopressin in the nanofibers was 8% (*w/w*). The weight of a 10 mm nanofiber disc (Figure 1b) was in the range of 1.5–3 mg; hence, the fiber discs provide a theoretical loading of desmopressin of 120–240 µg per disc, which is equivalent to the dose of desmopressin in the freeze-dried, sublingual tablets already on the market. Marketed freeze-dried tablets with desmopressin for sublingual administration (Minirin^®^ by Ferring Pharmaceuticals) contain 60, 120, or 240 µg of desmopressin per dose. Thus, clinically relevant doses of desmopressin per nanofiber patch were achieved.

### 2.2. Nanofiber–Film Hybrid System Ensures Controlled and Unidirectional Release of Desmopressin

**Figure 2:**
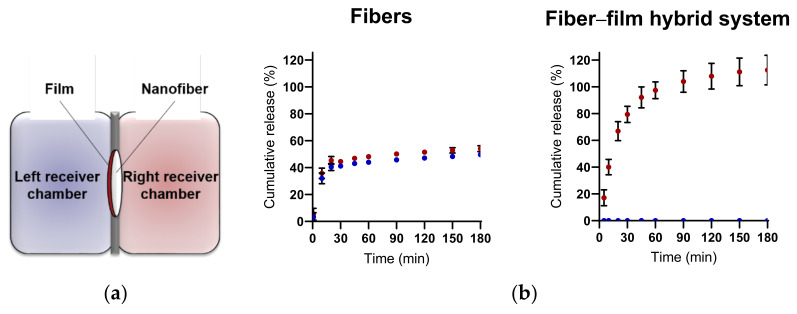
Nanofiber–film hybrid system facilitates unidirectional release of desmopressin. (**a**) Experimental setup used for studying the release of desmopressin from the nanofibers. The release of desmopressin into both chambers (in the Ussing chamber setup) separated either by the nanofibers or by the nanofiber–film hybrid system was determined. (**b**) Release of desmopressin from nanofibers or the nanofiber–film hybrid system in the left (blue) and right (red) Ussing chamber, respectively, as depicted in (**a**). The cumulative release was based on a loading of 8% (*w*/*w*) desmopressin in the chitosan/PEO nanofibers. The data are presented as mean ± SD. *N* = 3, *n* = 2–3, where *N* represent the number of individually prepared nanofibers/hybrid systems and *n* is the number of measurements per system.

A thin water-repelling backing film was applied to the mucoadhesive nanofiber layer to facilitate the unidirectional release of desmopressin, and, furthermore, to protect the drug delivery system and released therapeutic peptide from wash-out by saliva. The unidirectional and controlled release of desmopressin from the nanofibers and the barrier function of the water-repelling backing film were evaluated by determining the release of desmopressin into both sides of a diffusion cell (Ussing chamber) separated by the nanofiber–film hybrid system (Figure 2a). In the absence of the backing-membrane used as a control, approximately 50% of the loaded peptide was released from the nanofibers in the left and right chambers—i.e., it did not show a unidirectional release. We have previously demonstrated that electrospun chitosan/PEO nanofibers display extraordinary swelling properties and can swell >1000% (*w/w*) within 15 min of exposure to water [1]. Thus, a fast release of the highly water-soluble peptide desmopressin was therefore expected. Accordingly, approximately 80% of the encapsulated desmopressin was released fast (within 20 min) from the nanofibers, and the complete release of desmopressin, represented by the amount detected in both chambers, was observed after approximately 60 min (Figure 2b). In the presence of a water-repelling backing film, the unidirectional release of desmopressin from the nanofiber–film hybrid system was achieved, as <1% of the encapsulated desmopressin was detected in the left chamber fronting the backing membrane and approximately 80% of the loaded desmopressin was released within 45 min to the right receiver chamber fronting the nanofiber layer (Figure 2b). In total, 100% of desmopressin was released from the fiber–film hybrid system within 1 h (Figure 2b). This confirms that the nanofiber–film hybrid system indeed ensures the unidirectional and complete release of the encapsulated therapeutic peptide. The average cumulative release of desmopressin exceeded 100% after ≥90 min, but the cumulative release was not statistically significant different from 100% at any time point. A slight evaporation of the release medium over time at 37 °C can cause the average cumulative release of desmopressin to exceed >100% for some samples. The release of peptide was determined from 2–3 areas of the same electrospun nanofiber mat, and three individual nanofiber mats were evaluated. In general, no significant difference in peptide release was found between the individual areas nor mats, which is indicative of a homogenous distribution of peptide in the electrospun nanofibers.

### 2.3. Nanofiber–Film Hybrid System Protects Peptide from Wash-Out

**Figure 3:**
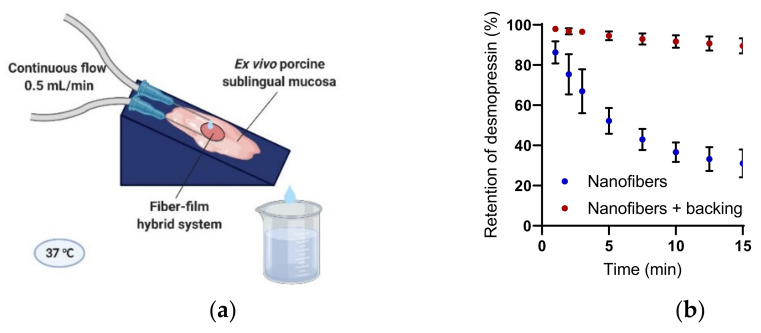
Nanofiber–film hybrid system protects desmopressin from wash-out by saliva. (**a**) Ex vivo porcine sublingual mucosa was mounted on a support with an angle of 16° and the retention of desmopressin on the tissue was evaluated over time. Created by Biorender.com. (**b**) Retention of desmopressin dosed in nanofibers with and without a saliva-repelling backing film on ex vivo porcine sublingual mucosa. *N* = 4, where N specifies the number of biological replicates.

Sublingual drug delivery is challenged by a continuous flow of saliva that leads to the wash-out of the drug delivery system and/or drug, which are subsequently swallowed. We have previously demonstrated the good mucoadhesive properties of electrospun chitosan/PEO nanofibers for the sublingual mucosa [8]. We hypothesize that a combination of the mucoadhesive properties of the electrospun chitosan-based nanofibers and the protective function of the backing film will facilitate the close adhesion of the nanofiber–film hybrid system to the sublingual region, and furthermore, protect desmopressin from wash-out by saliva. The ability of the nanofiber–film hybrid system to prohibit the wash-out of desmopressin was investigated by a flow retention model using ex vivo porcine sublingual mucosa (Figure 3a). The flow rate of whole saliva in humans is reported to be around 0.3 mL/min without stimulation and up to 7 mL/min during stimulation by mastication, etc. [16]. The flow rate used in this experimental setup was 0.5 mL/min, and thus was within the average salivary flow rate in vivo. Isotonic buffer with 0.05% (*w*/*v*) bovine serum albumin (BSA) with a pH of 6.8, which is the average pH of saliva secreted without stimulation [17], was used as the medium. The mucosa from the ventral side of the porcine tongue was chosen as the surface of adhesion as it, like the human sublingual mucosa, is non-keratinized, it has rete ridges, and its epithelium thickness is similar to that found in man [18,19].

MiniRin^®^ desmopressin freeze-dried tablets dissolved immediately when exposed to moisture, and thus the tablet disintegrated within seconds and was quickly washed off the ex vivo porcine sublingual mucosa after initiating the flow. The content of desmopressin released from the commercial tablets was not quantified due to the presence of gelatin in the tablets, which interfered with the analysis method. In contrast, the nanofibers, irrespective of the presence of a protective backing film, adhered strongly to the ex vivo porcine sublingual mucosa throughout the duration of the experiment. Desmopressin was quickly released and washed out of the nanofibers without a protective backing-film, al-though it was lost in significantly less time than desmopressin released from MiniRin^®^ freeze-dried tablets. Hence, the mucoadhesive nanofibers alone retained desmopressin significantly longer on the tissue in comparison to the marketed tablet. In accordance with the hypothesis, the nanofiber–film hybrid system further protected desmopressin from wash-out and retained approximately 90% of the loaded desmopressin on the tissue after 15 min of exposure to flow.

Mucoadhesive drug delivery systems, ensuring unidirectional release, thus not only limit loss of drug by wash-out, but also provide an advantageous increase in the residence time of the drug on the mucosal tissue, the site of absorption. Furthermore, a local environment with a high drug concentration can be achieved between the adhesive drug delivery system and the sublingual mucosa. This increases the concentration gradient of the drug across the mucosal barrier and can lead to improved absorption. Furthermore, the local treatment of, e.g., lesions in the oral mucosa is possible if wash-out is prevented. Site-specific drug delivery can limit exposure to other sites in the oral cavity in general, which is beneficial for drugs with an unpleasant taste and mouthfeel or teeth staining. Some drugs may indeed induce side-effects upon swallowing, which can be avoided if they are efficiently absorbed from the oral mucosa. Thus, electrospinning is an interesting approach to produce solid dosage forms for mucosal application, as this method benefits from short processing times, mild drying conditions and prospects for industrial scalabi-lity, continuous manufacturing, as well as reduced cost [20,21]. Additional benefits are that biopharmaceuticals, formulated as solid dosage forms, display improved drug stabi-lity and offer easier handling for patients or healthcare personnel, as well as a reduced cost of transportation [20].

## 3. Materials and Methods

### 3.1. Materials

Chitoceuticals chitosan 95/100 (degree of deacetylation 96%, Mw 100–250 kDa, chitosan-96) was purchased from Heppe Medical Chitosan (Halle, Germany). Polyethylene oxide (Mw 900 kDa, PEO), bovine serum albumin (BSA), acetic acid anhydride, Hank’s balanced salt solution (HBSS), Dulbecco’s phosphate buffered saline (PBS), glycerol (≥99%), tributyl citrate and ethyl cellulose, trifluoroacetic acid (TFA), and acetonitrile were obtained from Sigma Aldrich (St. Louis, MO, USA). N-2-hydroxyethylpiperazine-N’-2-ethanesulfonic acid (HEPES) was obtained from PanReac AppliChem (Darmstadt, Germany). Iron(III) oxide (Secovit^®^ E172) was from BASF (Copenhagen, Denmark). Desmopressin as TFA salt (purity > 98%) was obtained from SynPeptide (Shanghai, China). MiniRin^®^ freeze-dryed tablets-60 µg desmopressin (Ferring Pharmaceuticals, Copenhagen, Denmark) were purchased through the Association of Danish Pharmacies. Ultrapure water (18.2 MΩ × cm) purified by a PURELAB flex 4 (ELGA LabWater, High Wycombe, UK) was used.

### 3.2. Electrospinning of Chitosan/PEO Nanofibers with Desmopressin

**Figure 4:**
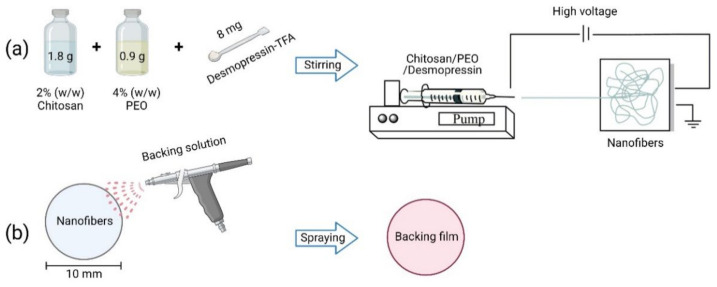
Preparation of the nanofiber–film hybrid system. (**a**) Electrospinning of chitosan/PEO na-nofibers with desmopressin as described in Section 3.2. (**b**) Spraying of film on nanofibers as described in Section 3.3. Created by Biorender.com.

The chitosan/PEO nanofibers were produced according to Stie et al., 2019 [7] (Figure 4a). Briefly, a 2% (*w*/*w*) clear solution of chitosan in 0.7% (*w*/*w*) acetic acid was prepared in ultrapure water and 4% (*w*/*w*) PEO was dissolved in ultrapure water. Both solutions were stirred for two days at room temperature (RT) to ensure the complete hydration of the polymers. To obtain a 1:1 (*w*/*w*) ratio of chitosan:PEO and a 8% (*w*/*w*) loading efficiency of the peptide, 1.8 g of 2% (*w*/*w*) chitosan and 0.9 g of 4% (*w*/*w*) PEO were mixed and stirred for at least 15 min, and hereafter, mixed with 8 mg desmopressin-TFA (corresponding to 6.6 mg desmopressin). The polymer-peptide solution was stirred for at least 30 min before electrospinning from a 1 mL syringe with a 20 G blunt needle (Photo-Advantage, Ancaster, ON, Canada) by an ES50P-10W high voltage source set to 20 kV. The temperature was controlled in the range 23–25 °C and a low humidity (<25% relative humidity) was maintained by a continuous flow of dry air. The nanofibers were collected on aluminum foil on a stainless steel collector plate placed 15 cm from the tip of the needle. The fibers were stored in a desiccator at 4 °C to avoid absorption of water and to preserve the stability of desmopressin. Further experiments were conducted within two weeks of the preparation of the electrospun nanofibers.

### 3.3. Preparation of Nanofiber–Film Hybrid System

Ten-millimeter discs of peptide-loaded nanofibers were punched out from the main mat and weighed to determine the exact loading of desmopressin per disc. Amounts of 141 mg of acetyl tributyl citrate, 47 mg glycerin, and 750 mg ethyl cellulose were dissolved in 15 mL ethanol (absolute) and stirred overnight at room temperature. Iron oxide was added and the solution was stirred for at least 30 min. The film was sprayed directly on the 10 mm discs of nanofibers employing an airbrush (Model BD-134, Custom Colors, Jyderup, Denmark) (Figure 4b). The fibers were fixed on an aluminum plate with holes under vacuum to avoid movement of the fibers as a result of the air flow from the airbrush during the application of the film-forming material by spraying.

### 3.4. Visualization of Nanofiber Morphology by Scanning Electron Microscopy

The morphology of the nanofibers was visualized by scanning electron microscopy (SEM), as previously described by Stie et al., 2019 [7]. The fiber–film hybrid system was cut with a scalpel and mounted vertically on the carbon tape to achieve an image of the cross-section of the fiber–film hybrid system. The average diameter of the nanofibers was determined using the Image J version 1.51j8 software (National Institute of Health, Bethesda, MD, USA) by measuring 25 individual fibers from four areas of interest from a total of three individual batches of electrospun nanofibers loaded with desmopressin.

### 3.5. Release of Desmopressin from Fiber–Film Hybrid System in Ussing Chambers

Discs of nanofibers (diameter 10 mm, weight 2–3 mg) or the nanofiber-film hybrid system were fixed in Ussing sliders with a diffusion area of 0.4 cm^2^ and placed in a side-by-side diffusion cells apparatus EM-CSY-8 Ussing chambers (Physiologic Instruments, San Diego, CA, USA), with 2 mL of 10 mM HEPES in HBSS pH 6.8 with 0.05% (*w*/*v*) BSA in each chamber, and incubated at 37 °C for 3 h. Samples of 100 µL were collected from each chamber at various time intervals and replenished with 100 µL warm 10 mM HEPES in HBSS pH 6.8 with 0.05% (*w*/*v*) BSA. The samples were centrifuged (10,000 rpm/9279× *g*, 10 min, 5 °C) and the concentration of desmopressin was determined by high-performance liquid chromatography with UV detection (HPLC-UV, Experimental Section 3.7). The cumulative release of desmopressin (M) was determined according to Equation (1).

(1) $$M=V_{S}\cdot \left(\sum_{n=1}^{n}C_{n-1}\right)+C_{n}\cdot V_{T}$$

where V_S_ is the sample volume (100 µL), C_n_ is the concentration of desmopressin at time point *n*, and V_T_ is the total volume of the receiver Ussing chambers (2 mL). The cumulative peptide release in percent (%) was calculated based on the theoretical loading of desmopressin of 8% (*w*/*w*) and a diffusion area of 0.4 cm^2^.

### 3.6. Ex Vivo Flow Retention Model

The ex vivo flow retention setup was inspired by Madsen et al. [17]. Sublingual porcine tissue from healthy pigs (approximately 30–60 kg, Danish Landrace/Yorkshire/Duroc) was collected immediately after euthanization and kept in PBS on ice until use on the same day as harvesting of the tissue. Thin sections (0.5–0.7 mm) of the ventral side of the tongue were obtained by means of an electric dermatome (Zimmer Biomet, Alberts-lund, Denmark). The sublingual mucosa was mounted on a rubber pad with pins at an angle of 16° and placed on a heating plate to achieve a temperature of approximately 37 °C of the tissue. The ex vivo sublingual mucosa was equilibrated for 10–15 min in warm (37 °C) 10 mM HEPES in HBSS pH 6.8 with 0.05% (*w*/*v*) BSA with a flow of 0.5 mL/min from two 13 G needles placed above the tissue and the flow was controlled by a syringe pump (11 Elite, Harvard Apparatus, Holliston, MA, USA). 10 mm discs of electrospun nanofibers, with or without backing, or a MiniRin^®^ freeze-dried tablet (60 µg desmopressin) were placed on the mucosal tissue and flushed with warm (37 °C) 10 mM HEPES in HBSS with 0.05% (*w*/*v*) BSA, pH 6.8 with a flow of 0.5 mL/min for 15 min controlled by a syringe pump (11 Elite, Harvard Apparatus, Holliston, MA, USA). Samples of 100 µL of the eluate were collected after 1, 2, 3, 5, 7.5, 10, 12.5 and 15 min and centrifuged (10,000 rpm/9279× *g*, 10 min, 5 °C). The concentration of desmopressin was determined by HPLC-UV (Experimental Section 3.7). As gelatin from the MiniRin^®^ tablets interfered with the HPLC-UV method, the concentration of desmopressin released from the freeze-dried tablets was not determined. The retention of desmopressin was determined according to Equation (2).

(2) $$\mathrm{Peptide}\;\mathrm{wash}\;\mathrm{out}\;\left(\mathrm{mg}\right)=V_{S}\cdot \sum_{n=1}^{n}C_{n-1}+C_{n}\cdot \left(V_{T}-\sum_{n=1}^{n}V_{n-1}\right)$$

where V_S_ is the eluate sample volume (100 µL), C_n_ is the concentration of desmopressin at time point *n*, V_T_ is the total volume of eluate at time point *n* based on a flow of 0.5 mL/min, and $\sum_{n=1}^{n}V_{n-1}$ is the sum of the eluate volumes sampled at time point *n* − 1. The retention of desmopressin in percent (%) was determined based on a theoretical loading of 8% (100% peptide retention) (*w/w*).

### 3.7. Quantification of Desmopressin by HPLC-UV

Desmopressin was quantified by HPLC-UV (λ218 nm) on a Shimadzu Prominence system (Kyoto, Japan) with an Aeris peptide XB-C18 column (100 × 2.1 mm, 3.6 μm, Phenomenex, Torrance, CA, USA). Desmopressin was eluted from 10 µL samples with a gradient 0→40% eluent B in eluent A over 8 min at 0.8 mL/min at 40 °C, where eluent A consisted of 95:5:0.1% (*v*/*v*) acetonitrile:water:TFA and eluent B of 5:95:0.1% (*v*/*v*) acetonitrile:water:TFA. The samples were stored at 4 °C during analysis. The limit of detection and limit of quantification were 0.09 µg/mL and 0.26 µg/mL, respectively.

## 4. Conclusions

The therapeutic peptide desmopressin was encapsulated within mucoadhesive electrospun chitosan/PEO nanofibers intended for sublingual delivery. A two-layered hybrid drug delivery system was developed by combining a saliva-repelling backing film with the nanofibers to ensure unidirectional drug release. The nanofibers displayed a unidirectional, controlled, and fast release of desmopressin with an approximately 80% release of the loaded peptide from the nanofiber-based hybrid system within 45 min. Importantly, the nanofiber–film hybrid system showed resilience to saliva flow and retained approximately 90% of the desmopressin loaded on the tissue after 15 min of exposure to flow. This can potentially improve the absorption of peptide, but also potentially improve the absorption of small molecular drugs across the mucosae. Mucoadhesive electrospun na-nofibers are considered promising carriers for peptide delivery via mucosal routes upon, e.g., sublingual administration.

## Data Availability

The data presented in this study are available on request from the corresponding author.
